# Biomechanical properties of periodontal tissues in non-periodontitis and periodontitis patients assessed with an intraoral computerized electronic measurement device

**DOI:** 10.1007/s00784-023-04859-w

**Published:** 2023-01-10

**Authors:** Karin Jepsen, Pia-Merete Jervøe-Storm, Isabel Henrichs, Ina Lensing, Alja Larissa Müller, Raluca Cosgarea, Ludger Keilig, Christoph Bourauel, Søren Jepsen

**Affiliations:** 1 Department of Periodontology, Operative and Preventive Dentistry, University Hospital Bonn, Welschnonnenstrasse 17, 53111 Bonn, Germany; 2 Clinic for Periodontology and Peri-Implant Diseases, Philipps University Marburg, Marburg, Germany; 3 Clinic of Prosthodontics, Iuliu Hatieganu University Cluj-Napoca, Cluj-Napoca-Napoca, Romania; 4 Oral Technology, University Hospital Bonn, Bonn, Germany; 5 Department of Dental Prosthetics, Propaedeutics and Materials Science, University Hospital Bonn, Bonn, Germany

**Keywords:** Tooth mobility measurement, Tooth displacement, Periodontitis, Periodontal ligament

## Abstract

**Objective:**

To identify tooth mobility (TM) by time-dependent tooth displacement using an electronic intra-oral loading device (ILD) in periodontally healthy and periodontally compromised patients.

**Materials and methods:**

Twenty-eight untreated periodontitis and 20 periodontally healthy patients [25 female and 26 male; ages: 20–81 years], contributing with 68 teeth (periodontitis: *n*_teeth_ = 28; non-periodontitis: *n*_teeth_ = 40), participated in the study. TM was measured in vivo by displacing central or lateral incisors to a maximum of 0.2 mm orally over durations of 0.5 s, 1 s, and 10 s with the ILD. The maximum force (Fmax) was extracted from the measured force/deflection curves for every single measurement.

**Results:**

Differences in TM-ILD values were found for periodontitis as compared to non-periodontitis patients derived from the same loading durations (differences of 3.9 (0.5 s), 3.1 (1 s), 2.8 (10 s), (95% CI for 0.5 s (1.2–6.7), *p* = 0.024; 1 s (1.4–6.0), *p* = 0.067; 10 s (0.2–5.3), *p* = 0.001), rejecting the null hypothesis of no difference (*T*-test) for durations of 0.5 and 10 s. There was a significant correlation of TM-ILD (Fmax) with BOP at 0.5 s (– 0.52) and with attachment loss at all time durations (– 0.47 at 0.5 s; – 0.57 at 1 s; – 0.47 at 10 s).

**Conclusions:**

This clinical investigation could demonstrate that time-dependent tooth displacements using a new computerized electronic device were associated with attachment loss and bleeding on probing.

**Clinical relevance:**

ILD can improve the monitoring of tooth mobility, as TM-ILD values reflect qualitative (inflammatory status interpreted by BOP) and quantitative parameters (interpreted as the amount of CAL loss) of periodontal disease.

**Supplementary Information:**

The online version contains supplementary material available at 10.1007/s00784-023-04859-w.

## Introduction

Periodontal tissues are complex structures, including the periodontal ligament (PDL), root cementum, alveolar bone, and attached gingiva. Biomechanical properties are complicated as tissues contain desmodontal fibers, blood vessels, nerves, and fluids filling in and/or linking the space between the root and the alveolar bone. These elements provide transverse and vertical intrinsic mobility to the root, and the elastic nature of collagen fibers and the dampening of the fluid phase is responsible for nonlinear and time-dependent behavior. The PDL is the most deformable tissue of the entire periodontium, so that tooth movements are possible under functional loading. Playing a decisive role in initial tooth movement [1], translations of 0.2 mm and rotations of 2° can be achieved. The initial tooth mobility depends on various factors, such as the number of roots and root anatomy, the width of the periodontal ligament, the distribution of the collagen fibers, the elastic properties of the surrounding tissues, as well as the loading velocities. With traumatic impact loads (high load velocities), the liquid phase may cause a strong dampening effect where the vascular network and the periodontal tissue fluid provide a hydrodynamic cushioning effect [2]. Up to now, occlusal trauma is a controversial subject in periodontology because it can only be confirmed histologically, and its clinical diagnosis still depends on clinical and radiographic surrogate indicators which make clinical trials difficult [3, 4]. Consequently, biomechanical characteristics are relevant to understand the behavior of the teeth under physiological loads in periodontal health and disease and how they change with an injury.

Vice versa, with various functions and abilities, the PDL transmits forces to the alveolar bone and is responsible for tissue remodeling [5]. It is well known that cells react to mechanical forces and convert mechanical cues to biochemical signals that are important also in the progress of orthodontic tooth movements. Here, the applied biomechanical forces differ significantly from physiological tooth movements resulting in an acute inflammatory response of periodontal tissues. A cascade of biological events is triggered with the synthesis and release of various neurotransmitters, arachidonic acid, growth factors, metabolites, cytokines, and enzymes relevant to the initiation of bone remodeling, which is usually not associated with a net loss of periodontal bone attachment [6] in the presence of untreated periodontitis; however, aggravation of biofilm-induced inflammation, irreversible tissue destruction may occur [7, 8].

Tooth displacement by physiological loading (chewing, swallowing, and clenching) depends on the direction and magnitude of the force, the root morphology, and the condition of the periodontium. Tooth mobility has been assessed by a large number of devices and methods by several investigators [9]. A large number of trials were performed using more sophisticated appliances, like the performance transducer [10], compressed air as the source of force [11], or Periotest® [12]. A major limitation of the latter device is that only dampening characteristics with a predefined frequency can be measured.

Recently, our group introduced an electronic measurement system that can record full force/crown deflection characteristics over a wide range of displacement velocities, from quasi-static loading to short-term pulses down to 0.5 s [2, 13, 14]. Consequently, the device has the capacity to noninvasively record tooth displacements with high resolution and to monitor the time-dependent biomechanical behavior of the PDL.

The aim of this in vivo study was to compare time-dependent tooth displacement/tooth mobility in periodontitis and non-periodontitis patients and to investigate the association between clinical attachment loss and bleeding on probing. The null hypothesis stated that there were no differences in the forces measured at 0.5, 1, and 10 s loading time at a deflection of 0.2 mm in health and disease.

## Material and methods

**Fig. 1:**
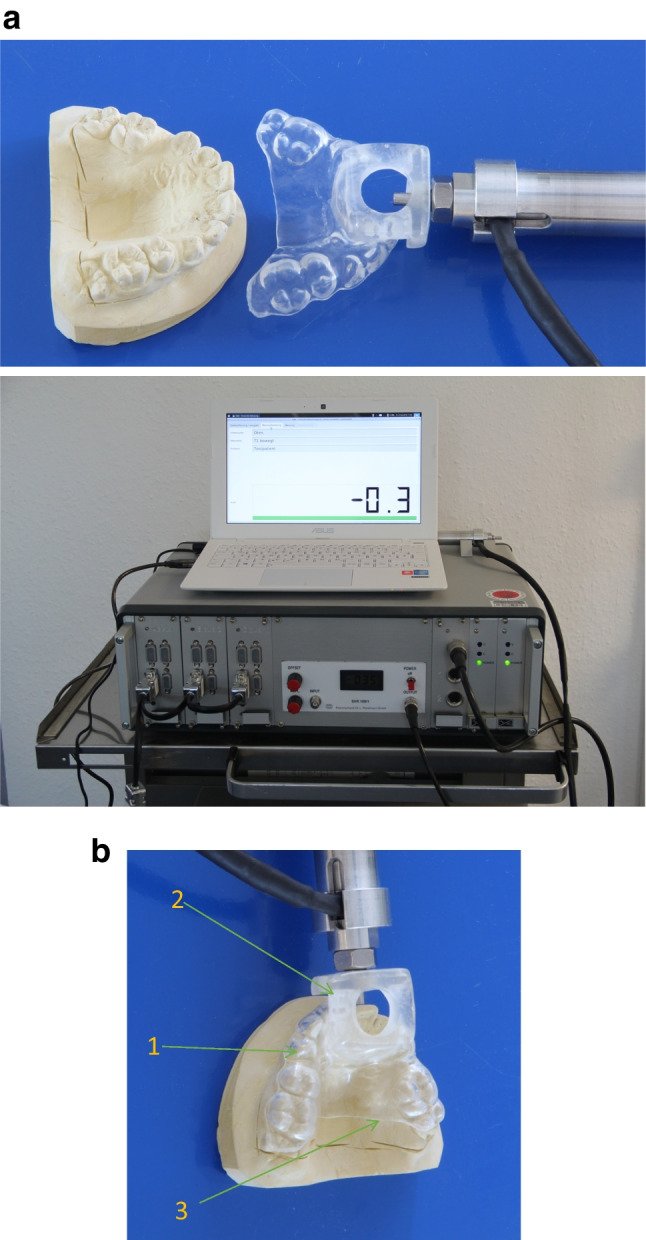
**a** Loading device (ILD) with a mounted splint connected to control electronics. **b** Individualized splint on a patient´s model consisting of a hard (outer) and soft layer (1), Adaptor for the loading device (2), dorsal part at the palate as short as possible (3)

This study was designed as a prospective observational, non-interventional clinical trial (ClinicalTrials.gov, identifier: NCT04646265) to investigate the biomechanical properties of the periodontal tissues in different populations with and without periodontal disease. The 3 observers (IH, IL, and LM)/investigators were properly trained on how to operate the intraoral loading device (ILD), how to adjust the measurement parameters, and how to use the software during patient measurements. The ILD is based on a system proposed by Yoshida et al. [15] and consists of a handpiece and a splint (Fig. 1a, b) to secure safe force application.

Study participants were consecutively recruited from patients in Germany (University of Bonn). Ethics approval was obtained by the Ethical Committee, University of Bonn (record ethics #030/12, updated in 2020). All subjects gave their informed consent after the investigators had provided a thorough explanation of the study procedures and their associated risks and benefits; all study procedures were performed according to the Declaration of Helsinki (1975, revised in 2013) on experimentation involving human subjects.

Individuals who fulfilled the following inclusion and exclusion criteria were invited to participate:

### Inclusion criteria

Patients with periodontitis [16]:

- Intact anterior upper or lower dentition
- No trauma from occlusion
- Voluntarily signed informed consent form before any study-related procedures
- At least 18 years of age

Periodontally healthy participants [17] with

- FMBS (full mouth bleeding score) < 10% [18]
- FMPS (full mouth plaque score) < 25% [19]

### Exclusion criteria

Patients with:

- Uncontrolled diabetes or other uncontrolled systemic diseases
- Disorders or treatments that compromise wound healing
- Medical conditions requiring chronic high-dose steroid therapy
- Bone metabolic diseases
- Radiation or other immuno-oppressive therapy
- Presence of oral lesions (e.g., ulceration, malignancy)
- Mucosal diseases (e.g., lichen planus, mouth ulcer)
- History of malignant disease in the oral cavity or previous radiotherapy to the head or neck
- Inadequate oral hygiene or unmotivated for adequate home care
- Female subjects who are nursing, pregnant, or plan to become pregnant
- Antibiotic treatment in the previous 3 months

## Measurements

### Tooth selection

In order to minimize bias, in case of more than four incisors suitable for biomechanical evaluation, a maximum of two were randomly assigned using a random number generator.

### Clinical measurements

Periodontal health status at the test teeth was assessed using the following recordings at six sites/tooth (mesiobuccal, buccal, and distobuccal and mesio-oral, oral, and disto-oral):

- Plaque index (PI): dichotomous decision 0/1
- Bleeding on probing (BOP): dichotomous decision 0/1
- Probing pocket depth (PPD) (mm)
- Clinical attachment level (CAL) (mm)

The measurements of CAL and PPD were obtained with a pressure-sensitive probe (Click-Probe, Kerr, Switzerland) to the nearest millimeter at six sites per tooth. Bleeding on probing (BOP) was assessed dichotomously (as present or absent), and BOP was positive if it occurred within 15 s after periodontal probing.

### Tooth mobility

- Mobility grades (1–3) were conventionally assessed according to Miller [20].
- For tooth mobility measurements with the intraoral loading device (TM-ILD) (Fig. 1a), individualized splints consisting of a hard and soft layer (Fig. 1b) were pre-fabricated for each patient as previously described [2]. Measurements of a preselected tooth were taken by displacing the center of the anatomic crown in a labio-lingual direction. Simultaneously, the resulting forces were recorded. During the loading phase, the tooth was displaced linearly from zero up to 0.2 mm over a time period of 0.5 s, 1 s, or 10 s. During the unloading phase, tooth displacement was reduced back to zero. A minimum pause of 1 min was given after each measurement for relaxation of the PDL, normalization of the hydrodynamics of the fluid phase, and realignment of the tooth to its initial position. Displacements of teeth were restricted to 0.2 mm, a built-in feature of the ILD. All measurements were repeated after the first measurement, at least 6 h apart. Duplicate measurements were performed by the same operator.

### Statistical analysis

Descriptive statistics were summarized as means and standard deviations for quantitative data and percentages for qualitative data. Means for each treatment group and differences between treatment groups were presented, along with associated 95% confidence intervals as well as *p*-values for differences between groups. The null hypothesis was that there were no differences in TM-ILD values between groups.

For the assessment of biomechanical properties of the periodontium, clinical chairside data were collected, and computerized measurements of TM-ILD generated from the ILD were allowed for export via Excel into the statistical software program using the software PRISM version 8.4.3 (GraphPad Inc., Software, USA). Prior to statistical analysis, the maximum force (*F*max in N/mm) was extracted from the measured force/deflection curves for every single measurement. Quantitative descriptive statistics were performed to calculate the means of Fmax and standard deviations for each loading duration and each patient group measured.

Comparisons between periodontitis and non-periodontitis-affected maxillary teeth and location (maxillary versus mandibular) were performed using the Kolmogorov–Smirnov test and Welch’s unequal variances *t*-test for comparisons between groups. An *α* error of 0.05 was set to accept a statistically significant difference. The reproducibility of measurements and the association of TM-ILD values with CAL (mm) as well as BOP (%) were evaluated by Spearman rank correlation testing.

## Results

Between November 2020 and February 2021, a total of 52 patients were consecutively recruited and screened. Twenty-eight periodontitis-affected incisors (28 patients) were selected as the test, and 40 teeth in 20 healthy patients serving as control were included in the study. Patient characteristics are listed in Table 1. In the periodontitis group, the maximum CAL loss ranged between 4 and 15 mm, maximum PPD was 10 mm. The mean BOP-score for the target teeth calculated out of 6 sites/tooth amounted to 36.9 ± 32.8% in periodontitis-affected teeth. In periodontal health, mean BOP scores were 1.7 ± 6.2% in the maxillary and 2.6 ± 7.4% in the mandibular incisors.

**Table 1:** Patient and target tooth characteristics

	Periodontitis patients *N*** = **28 (*n*_teeth_ = 28)	Periodontally healthy participants *N* = 20 (*n*_teeth_ = 40)
Age (years); (mean ± SD) (Range)	53.8 ± 14.4 (range: 21–81)	26.0 ± 3.4 (20–31)
Male (*n*)	15 (54%)	10 (50%)
Female (*n*)	13 (46%)	10 (50%)
Periodontitis		
***Stage I/II/III/IV (*n*)	0/3/20/5	N/A
Grade A/B/C (*n*)	0/16/12	N/A
FMPS mean %	36.9 ± 21.4	10.5 ± 7.3
FMBS mean %	34.5 ± 19.7	2.7 ± 2.0
Smoking status		
Current smokers (*n*)	22	2
Nonsmoker (*n*)	6	18
Maxilla		
Central/lateral incisor (*n*)	23/5	8/12
Mandible		
Central/lateral incisor (*n*)	0	19/1
Target tooth		
Clin. attachment level (CAL) (mm)* mean ± SD	6.9 ± 2.7 (range 4–15)	No attachment loss/no bone loss
Probing pocket depth (PPD) (mm), mean ± SD*		
Maxilla	5.9 (range 4–10)	1.9 ± 1.7
Mandible		1.8 ± 1.7
Bleeding on probing (BOP) (%)** Mean ± SD		
Maxilla	36.9 ± 32.8	1.7 ± 6.2
Mandible		2.6 ± 7.4
Mobility		
0/1/2/3 (Miller 1938)| *n*_teeth_	15/12/1/0	40/0/0/0

^*^Target tooth mean; ** BOP out of 6 sites per (target) tooth; ***Papapanou et al. [16]; *FMPS*, full mouth plaque score [19]; *FMBS*, full mouth bleeding score[18]

**Fig. 2:**
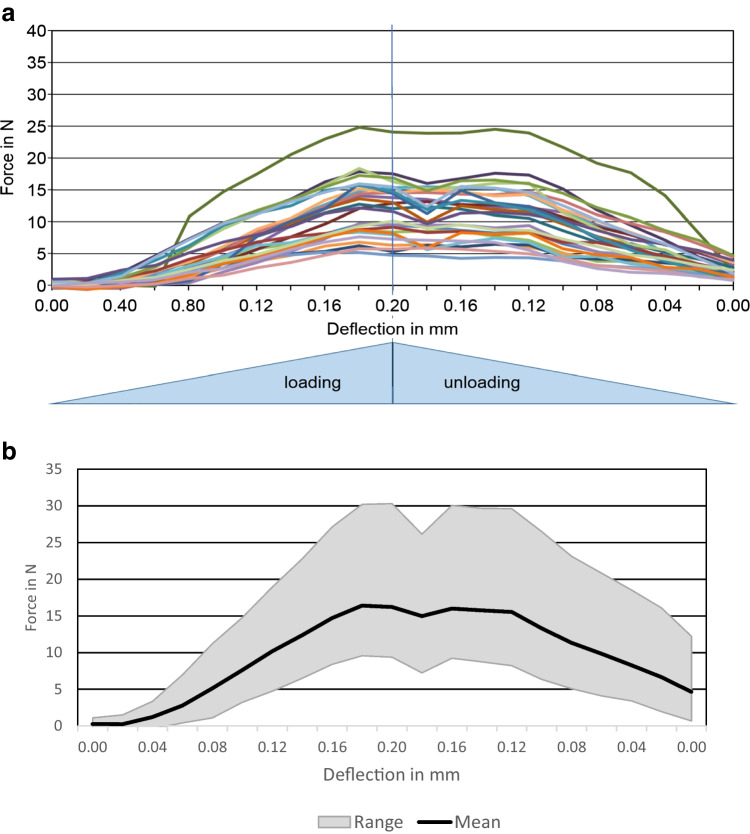
**a** Exemplary force/crown deflection curves up to a maximum of 0.2 mm to the lingual for each periodontitis patient (loading duration 0.5 s). **b** Exemplary force/crown deflection ranges up to a maximum of 0.2 mm to the lingual for each periodontally healthy patient, duplicate measurements included (loading duration 0.5 s)

In vivo measurements of tooth mobility revealed large inter-individual differences for the three investigated time points. Examples of crown deflection curves with a loading duration of 0.5 s are shown for periodontitis-affected teeth (Fig. 2a) and ranges with a mean curve for healthy patients in Fig. 2b.

Comparing healthy maxillary and mandibular incisors, statistically, significant differences were found at 10 s (*p* = 0.011, CI: (0.7–4.5)) (Supplementary Table 1). *F*max measured on all maxillary incisors after displacement (ILD) with different loading durations (0.5 s, 1 s, and 10 s) are shown in Table 2. Irrespective of loading times, the amount of Fmax was similar (0.5 s: 16.3 ± 6.3; 1.0 s: 15.4 ± 6.6; 10 s: 16.3 ± 6.3). In periodontally compromised teeth *F*max from longer loading times (10 s) with 10.1 ± 5.1 were lower than short-duration forces (12.3 ± 5.2; 12.4 ± 4.6).

**Table 2:** Mean maximum force values ± standard deviations calculated from the tooth mobility measurements (TM-ILD) with loading duration in seconds (0.5 s, 1 s, and 10 s) for all patients (*n* = 48). Measurements for non-periodontitis (*n* = 20) versus untreated periodontitis patients (*n* = 28) maxillary incisors were compared by *t*-test (*Welch’s unequal *t*-test, followed by Bonferroni–Holm correction)

	Healthy participants Mean** ± **SD (95% CI)	Periodontitis patients Mean** ± **SD,(95% CI)	∆ Health-periodontitis Mean** ± **SD (95% CI)	*T*-test unpaired *t*-test*
	20 teeth/ 20 patients	28 teeth/ 28 patients		
Maximum force/displacement (TM-ILD) [N/mm]				
0.5 s	16.3 ± 6.3 (13.4–19.3)	12.4 ± 4.6 (10.5–14.2)	3.9 ± 1.4 (1.2–6.7)	*p* = 0.024
1.0 s	15.4 ± 6.6 (12.3–8.5)	12.3 ± 5.15 (10.2–14.3)	3.1 ± 1.5 (1.4–6.0)	*p* = 0.067 (NS)
10 s	16.3 ± 6.3 (13.4–19.3)	10.1 ± 5.1 (8.1–12.1)	2.8 ± 1.3 (0.2–5.3)	*p* = 0.001

A comparison between data from the same loading durations for periodontitis and healthy patients (maxillary incisors) revealed a significant difference of 3.9 (0.5 s) and 2.8 (10 s), a 95% confidence interval for 0.5 s (1.2–6.7; *p* = 0.024) and 10 s (0.2–5.3, *p* = 0.001), formally rejecting the null hypothesis of no difference in TM-ILD values between these two groups (Table 2).

Correlation-coefficients (Spearman rank) of repeated measurements of *F*max (0.5 s, 1.0 s, and 10 s) varied between 0.79 and 0.68, indicating good reproducibility of measurements (Table 3). Rank correlation analysis also demonstrated that TM-ILD values were primarily related to attachment loss (Table 4). The other clinical parameter BOP tested was related to the TM-ILD measurements at 0.5 s (Table 5).

**Table 3:** Correlation [Spearman rank] of repeated measurements with the ILD

Repeated measurements	Max force/displacement (TM-ILD) [N/mm]		
	0.5 s	1.0 s	10 s
Spearman rank			
*R-*coefficient of correlation	0.68	0.79	0.74
95% confidence interval	0.45 to 0.82	0.52 to 0.91	0.43 to 0.89
*P*-value (two-tailed)	0.0001	0.0001	0.0001

**Table 4:** Negative association [Spearman rank] between attachment loss and *F*max

CAL (%) relationship to	*F*max (ILD) [N/mm]		
	0.5 s	1.0 s	10 s
Spearman rank			
*R*-coefficient of correlation	-0.47	-0.57	-0.47
95% confidence interval	-0.72 to -0.092	-0.79 to -0.23	-0.72 to -0.10
*P*-value (two-tailed)	0.0143	0.0019	0.0122

**Table 5:** Negative association [Spearman rank] between BOP and *F*max at loading time 0.5 s

BOP(%) relationship to	Max force/displacement (ILD) [N/mm]		
	0.5 s	1.0 s	10 s
Spearman rank			
*R*-coefficient of correlation	− 0.52	− 0.27	− 0.34
95% confidence interval	− 0.76 to − 0.16	− 0.60 to 0.14	− 0.64 to 0.047
*P*-value(two-tailed)	0.0057	0.176	0.0748

## Discussion

The present study was designed to test the hypothesis that biomechanics of the PDL, as assessed with an electronic intraoral loading device, is different in untreated periodontitis patients compared to healthy patients. Our results could confirm for the first time that there were indeed significant differences in TM-ILD values for the teeth of patients with periodontal health and disease. Furthermore, we investigated the association of tooth mobility as expressed by TM-ILD values with clinical attachment loss and bleeding on probing and could establish a significant inverse negative correlation with these parameters reflecting quantitative (CAL) and qualitative (BOP) measures of periodontitis.

The results showed also that the customized loading device for in vivo measurements of TM-ILD values demonstrated good reproducibility of mean Fmax measurements for healthy patients. These findings confirm an investigation of repeatability, which was performed using an idealized maxillary model simulating the PDL using silicone [13]. Here, the variation of the forces determined by repeated measurements was below 5%.

Fmax resulting from longer loading times (10.0 s) appeared to be lower than from short durations in this study in periodontitis patients (Table 2). This is in agreement with previous studies [13, 21] using the same measurement device. The difference in force levels between the different velocities observed in the periodontitis group is probably caused by the multiphasic composition of the periodontal ligament. The fluids contained in the PDL act as a damper, resulting in a stiffer behavior (higher forces) on fast loading and softer (lower forces) on slow loading.

Konermann et al. [14] recorded tooth mobility upon displacement (TM-ILD) during retention after fixed multibracket appliance therapy. In young patients, a similar pattern was found 6 months after debonding. Measurements were also conducted on incisors and during the loading phase over a time period of 0.2, 0.5, 1, 2, 5, and 10 s with linear tooth displacement from zero to maximum displacement of 0.2 mm. *F*max was calculated to be 19.8 (± 6.9) (0.5 s), 19.6 (± 7.2) (1 s) 16.0 (± 6.4) (10.0 s). The difference to our study population was that their patients were younger [mean age 16.1 ± 3.1 years] than ours, with a mean age of 26 years. However, in the study by Konermann et al., [14] the inflammatory status of the investigated teeth was not clearly defined, whereas we were able to examine healthy patients according to the current classification of periodontal diseases [17, 22]. Notably, in our healthy participants, Fmax was calculated to be 16.3 (± 6.3) (0.5 s), 15.4 (± 6.6) (1 s) 16.3 (± 6.3) (10.0 s) representing a predefined state of health associated with no attachment-loss and minimal bleeding on probing.

In general, the magnitude of forces that can be observed in the oral cavity covers a very large range. For orthodontic treatment (slow loading), forces below 1 N are typically recommended [23]. For voluntary molar biting forces and clenching (fast loading), mean forces above 500 N can be found in the literature [24, 25]. The loading velocities used in our study lie between the fast loading of the teeth during chewing and the almost static loading in orthodontic treatment, and our measured Fmax values are at the lower end of the clinically observed force range.

Previous research on tooth mobility using compressed air as the source of force could demonstrate that tooth mobility in subjects with healthy periodontal conditions was consistently lower than the mobility of the teeth in subjects with periodontal disease [11]. These results were confirmed by Schulte et al. [12], who found a strong association with radiographic bone loss using Periotest® measurements, whereas the authors found no correlation to clinical measures of a papillary hemorrhagic index or PPD.

We also evaluated aspects of the inflammatory status interpreted by BOP and could show that BOP was related to TM-ILD values at short loading times of 0.5 s using our device. This is in agreement with other authors who demonstrated a reduction of TM assessed by compressed air as the source of force concomitant with the gingival improvements [26].

Tooth mobility, or tooth displacement under physiological loading, is an important factor in periodontal disease. One limitation of our study was that our patient population did not exhibit high variability in TM as assessed traditionally. In consequence, correlation analyses between the Miller grading system [20] and TM-ILD values were not meant to perform. Other limitations were the lack of information on the inter-observer reproducibility and the fact that the majority of periodontitis patients were smokers, whereas only two of the participants in the healthy group did smoke. Smoking is one of the major etiologic factors of periodontitis [4]. Therefore, it is not surprising that the majority of patients in the periodontitis group were smokers. The relative impact of smoking could not be discerned in the present study. However, this is an interesting topic for future studies.

The present data will serve to further develop our device in the future. At present, the deflection of the tooth is measured at the contact point of the thrust die. A full three-dimensional reconstruction of the movement using hall sensors and magnets integrated into the ILD is planned but has not yet been calibrated and installed. Thus, a 3D movement reconstruction could only be realized by subsequent finite element simulations using patient-individualized models. Such simulations are planned, based on the patient data registered during this study.

## Conclusion

Among multiple factors determining tooth prognosis in patients undergoing treatment for periodontitis, tooth mobility (clinically assessed), and bone loss at baseline have been identified as significant predictors for tooth loss [27]. The ILD used in this study records noninvasively full force/deflection characteristics of teeth over a wide range of displacement velocities, from quasi-static loading to short-term pulses down to 0.5 s. Our TM-ILD values reflected qualitative (inflammatory status interpreted by BOP) and quantitative factors (interpreted as the amount of CAL loss) of periodontal disease. In the future, our device might be used to monitor the course of disease more objectively and may help to develop more accurate models for tooth survival in patients treated for periodontitis in order to make treatment more predictable.

## Supplementary Information

Below is the link to the electronic supplementary material.

Supplementary file1 (DOCX 26 KB)
