# Supplementary material for: Biomechanical properties of periodontal tissues in non-periodontitis and periodontitis patients assessed with an intraoral computerized electronic measurement device
Source: Clin Oral Investig. 2023 Jan 10;27(2):797–805. doi: 10.1007/s00784-023-04859-w (PMC9889448; doi:10.1007/s00784-023-04859-w)
Supplement: Supplementary file 1 — Supplementary file1 (DOCX 26 KB) [file 784_2023_4859_MOESM1_ESM.docx]

**Supplementary table Table 1** Mean maximum force values ± standard deviations calculated from the tooth mobility measurements (TM-ILD) with loading duration in seconds (0.5 s, 1 s, 10 s) for healthy participants (n = 20). Measurements for mandibular (n=20) versus maxillary incisors (n=20) in the same patients were compared by paired t-test.

|  | **Healthy participants**  **mean ±SD, (95% CI)** | | **∆ Maxilla-Mandibula**  **Mean, (95% CI)** | **Paired T-test** |
| --- | --- | --- | --- | --- |
|  | 20 Incisors (maxilla)  20 Patients | 20 Incisors (mandibula)  20 Patients |  |  |
| **maximum force /displacement (TM-ILD)**  **[N/mm]** |  |  |  |  |
| 0.5 s | 16.3±6.3  (13.4-19.3) | 15.9±4.6  (18.1-13.7) | 0.40±4.2  (-1.6-2.4) | NS |
| 1.0 s | 15.4±6.6  (12.3-18.5) | 15.7±6.0  (18.5-12.8) | -0.29±5.0  (-2.6-2.0) | NS |
| 10 s | 16.3±6.3  (13.4-19.3) | 13.7±6.1  (16.6-10.9) | 2.6±4.1  (0.7-4.5) | p = 0.011 |
|  |  |  |  |  |
